# Supplementary material for: Complex‐centric proteome profiling by SEC‐SWATH‐MS
Source: Mol Syst Biol. 2019 Jan 14;15(1):e8438. doi: 10.15252/msb.20188438 (PMC6346213; doi:10.15252/msb.20188438)
Supplement: Supplementary file 8 — Dataset EV7 [file MSB-15-e8438-s008.zip › feature_plots_string/Q03701.pdf]

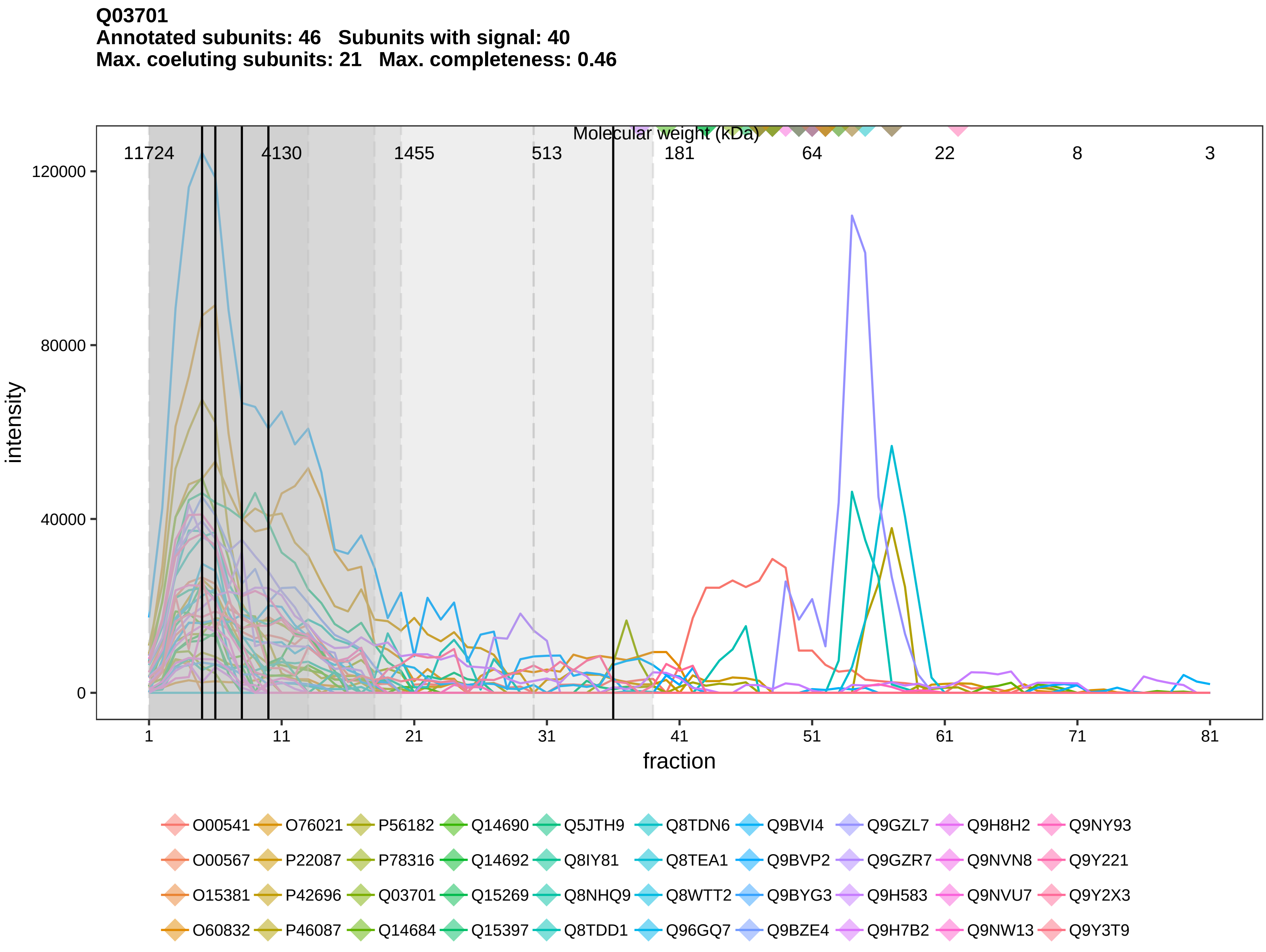

Q03701
Annotated subunits: 46 Subunits with signal: 40
Max. coeluting subunits: 21 Max. completeness: 0.46
Molecular weight (kDa)
11724
4130
1455
513
181
64
22
8
3
120000
80000
40000
0
intensity
1
11
21
31
41
51
61
71
81
fraction
O00541
O76021
P56182
Q14690
Q5JTH9
Q8TDN6
Q9BVI4
Q9GZL7
Q9H8H2
Q9NY93
O00567
P22087
P78316
Q14692
Q8IY81
Q8TEA1
Q9BVP2
Q9GZR7
Q9NVN8
Q9Y221
O15381
P42696
Q03701
Q15269
Q8NHQ9
Q8WTT2
Q9BYG3
Q9H583
Q9NVU7
Q9Y2X3
O60832
P46087
Q14684
Q15397
Q8TDD1
Q96GQ7
Q9BZE4
Q9H7B2
Q9NW13
Q9Y3T9
